# HGK promotes metastatic dissemination in prostate cancer

**DOI:** 10.1038/s41598-021-91292-2

**Published:** 2021-06-10

**Authors:** Sara Garcia-Garcia, Maria Rodrigo-Faus, Noelia Fonseca, Sara Manzano, Balázs Győrffy, Alberto Ocaña, Paloma Bragado, Almudena Porras, Alvaro Gutierrez-Uzquiza

**Affiliations:** 1 Departamento de Bioquímica y Biología Molecular, Facultad de Farmacia , Universidad Complutense de Madrid, Ciudad Universitaria , Madrid, Spain; 2 Instituto de Investigación Sanitaria del Hospital Clínico San Carlos (IdISSC), Madrid, Spain; 3 Department of Bioinformatics and 2nd Department of Pediatrics, Semmelweis University, Budapest, Hungary; 4 TTK Cancer Biomarker Research Group, Budapest, Hungary

**Keywords:** Bone metastases, Prostate cancer, Metastasis, Genetic engineering

## Abstract

Metastasis is the process of cancer cell dissemination from primary tumors to different organs being the bone the preferred site for metastatic homing of prostate cancer (PCa) cells. Prostate tumorigenesis is a multi-stage process that ultimately tends to advance to become metastatic PCa. Once PCa patients develop skeletal metastases, they eventually succumb to the disease. Therefore, it is imperative to identify essential molecular drivers of this process to develop new therapeutic alternatives for the treatment of this devastating disease. Here, we have identified *MAP4K4* as a relevant gene for metastasis in PCa. Our work shows that genetic deletion of *MAP4K4* or pharmacological inhibition of its encoded kinase, HGK, inhibits metastatic PCa cells migration and clonogenic properties. Hence, *MAP4K4* might promote metastasis and tumor growth. Mechanistically, our results indicate that HGK depleted cells exhibit profound differences in F-actin organization, increasing cell spreading and focal adhesion stability. Additionally, HGK depleted cells fails to respond to TNF-α stimulation and chemoattractant action. Moreover, here we show that HGK upregulation in PCa samples from TCGA and other databases correlates with a poor prognosis of the disease. Hence, we suggest that it could be used as prognostic biomarker to predict the appearance of an aggressive phenotype of PCa tumors and ultimately, the appearance of metastasis. In summary, our results highlight an essential role for HGK in the dissemination of PCa cells and its potential use as prognostic biomarker.

## Introduction

According to the World Health Organisation, Prostate cancer (PCa) is the second most frequent cancer among men and the fifth leading cause of all deaths^1^. Although most PCa patients have a high survival rate, the 5-year survival rate for patients who develop Castration-Resistant Prostate Cancer (CRPC) or metastasis is 29%. CPRC is the result of a prostate tumorigenesis process where low-grade prostatic intraepithelial neoplasia (PINs) progresses to an aggressive adenocarcinoma that does not respond to hormone deprivation therapy. During this process the tumor can spread to distant organs becoming a metastatic disease^2^. In fact, around 80% of CRPC patients will evolve to metastatic CRPC^1^. Indeed, 6% of men diagnosed with PCa will develop metastatic CRPC for which effective treatment has not yet been developed. Transition to CRPC is the main, but not the only, source of metastatic dissemination, since 3% of PCa patients will have metastatic dissemination at the time of diagnosis^2^.

Metastasis is the process that allows cancer cell dissemination from the primary tumor to other organs in the body and their seeding in this new niche, generating new tumors. The vast majority of PCa metastasis are found in the perivascular niches of the bone marrow sinusoids, which seems to be the preferred metastatic niche of PCa tumor cells^3^. The molecular mechanisms beneath cell migration movements of tumoral cells are complex, heterogeneous and poorly understood, limiting the development of effective therapies. Nonetheless, it is accepted that the occurrence of this process involves rearrangements of cell cytoskeleton and altered signalling of cell adhesion receptors leading to improvement of motility and invasive properties in tumor cells^4^. Therefore, the inhibition of any of these processes has the potential to prevent the metastatic event.

Protein kinases represent a promising group to explore due to its ability to regulate signaling pathways involved in cell motility and its susceptibility of being inhibited by chemical compounds. Several studies have associated the aberrant expression of these proteins with PCa metastasis^3^. In an interesting study, Falteimer et al., identified 20 kinases with metastatic promoting ability^4^, including several members of the MAPK superfamily.

HGK is a member of the STE20 family and a component of the MAPKs pathway. It is a serine/threonine kinase reported to be activated by TNF-α as an upstream regulator of the stress-induced c-jun-N-terminal (JNK) signaling pathway and activating transcription factor 2 (ATF2)^5^. Emerging evidences suggest that HGK regulates many cellular functions that control cell motility, proliferation, wound healing and stress^6^. In normal tissues, higher levels of *MAP4K4* (gene encoding HGK protein) mRNA are found in brain and in testis, and notably, it is markedly expressed in 40 out of 60 cancer cells lines derived from a variety of tissues^7^. Previous studies have shown associations between HGK levels and cancer. For example, HGK protein levels were elevated in lung adenocarcinoma^8^, pancreatic cancer^9^, hepatocellular carcinoma^10^, kidney tumors and brain tumors^7^. In the same line, upregulation of its expression was associated with a poor patient prognosis in colorectal cancer^11^, HCC^10^, pancreatic ductal adenocarcinoma^12^ and lung adenocarcinoma^13^.

Interestingly, high levels of *MAP4K4* expression and/or protein levels augmented proliferation and invasion in ovarian cancer^6,14^, medulloblastoma^15^, and glioblastoma^16^. HGK is also involved in actin dynamic regulation and has been shown to facilitate migration in keratinocytes^17^. Different hypothesis have been proposed to explain the molecular mechanisms by which HGK may regulate cancer cells disseminative properties; such as the upregulation of the *MYC* oncogene^18^, the MAPKs^8^ or the induction of the epithelial to mesenchymal transition in glioblastoma^19^. However, the proposed molecular mechanisms are not consistent across the different studies and none of them consider its role as an actin dynamic regulator.

Understanding the molecular mechanisms and signaling pathways that drive migration and invasion in PCa may lead not only to find better biomarkers of tumor status, but also novel therapeutic targets for the treatment of this disease. Due to the high number of functions that this protein is able to coordinate, we hypothesized that HGK functions in PCa cells as a hub, integrating growth factor and stress signals in a way that could promote motility and invasiveness. In order to test this hypothesis and shed some light into the molecular mechanisms used by HGK to regulate the metastatic process of prostate cancer, we have analyzed the role of HGK in PCa cells by applying the novel CRISPR/Cas9 knock out approach.

## Material and methods

### Patient cohort and RNAseq analysis

Publicly available level 3 normalized RNAseqV2 data of 537 prostate adenocarcinoma (PRAD) patients from TCGA was downloaded from FIREBROWSE (http://firebrowse.org/) and CICbioGUNE (https://www.cicbiogune.es/). Samples were classified in three groups: solid normal tissue, primary tumor and metastasis. U-Mann–Whitney statistical test was applied to check whether there were differences in *MAP4K4* gene expression between the three groups of patients.

### Cells culture maintenance and reagents

LnCaP and 22rv1 PCa cell lines obtained from ATCC (Manassas, VA, USA) were kindly donated by Dr. Guillermo Velasco. PC3 and DU145 cell lines were obtained from ATCC (Manassas, VA, USA). LnCaP, 22rv1 and DU145 were cultured in RPMI 10% FBS. PC3 were cultured in RPMI/F12 10% FBS. HEK-293 T cells cultured in 10% FBS DMEM medium were used for virus amplification. To trigger HGK signaling cells were treated with TNF-α (20 ng/mL; R&D, Minneapolis, MN, USA). The chemical inhibitor used to inhibit HGK was PF 06,260,933 (1–10 µM; Sigma, PZ0272).

### RNA Isolation, Reverse Transcription-PCR, and q-PCR analysis

Total RNA was isolated from the cells using RNeasy Mini Kit (QIAGEN, Hilden, Germany). Reverse transcription PCR (RT-PCR) was performed using Supercript IV Reverse Transcriptase (ThermoFisher, Waltham, MA, USA). Triplicate samples with their corresponding controls were assessed by qPCR, performed at the Universidad Complutense de Madrid (UCM) Genetic and Genomic facility. Melting curves of all qPCR reaction were closely monitored to avoid non-specific SYBR green signals. Gene fold changes were determined by the 2-ΔΔCt algorithm^20^. All primer sequences used are listed in supplementary section. GAPDH was applied as reference gene to normalize the 2-ΔΔCt based assessments and LNCaP was used to normalize the expression between cell lines.

### Protein expression

Cells were lysed in RIPA buffer with protease and phosphatase inhibitors (PMSF 1 mM, aprotinin 10 μg/ml, leupeptin 10 μg/ml, Na_3_VO_4_ 1 mM y NaF 20 mM). Protein concentrations were measured by BCA method using a standard curve prepared with known concentrations of BSA. Samples were prepared using Laemmli 4 × buffer and incubating for 5 min at 95 °C. Equal concentration of protein was adjusted with RIPA buffer. SDS-PAGE gels were transferred to PVDF membranes, blocked with 5% not fat milk in Tween 20 Tris-buffered saline (TTBS) for 1 h and then incubated with the following primary antibodies 1:1000 diluted in 5% BSA TTBS overnight. Actin CST-#3700, Tubulin CST#3873, HGK CST-#5146, P-p38 CST-#9211, P-FAK CST-#3283 and P-SAPK/JNK CST-#9251 were used as primary antibodies. Membranes were washed with TTBS and incubated with secondary anti-mouse IgG SCT sc-#516102 or Anti-rabbit IgG SCT sc-#237 antibodies conjugated with HRP in 5% BSA TTBS for 1 h (1:5000). HRP luminescence was stimulated using Clarity Western ECL Substrate (Bio-Rad) and detected with VWR image view station. ImageJ was used for quantification.

### CRISPR/Cas9 depletion cells production

Lenti-Cas9 Viral production was performed in 4 × 10^5^ HEK-293 T cells seeded in 10 cm tissue culture plates coated with 0.2% gelatine (skin porcine). A mixture of the 3 transfection plasmids (Packaging and enveloping plasmids psPAX2 and pMD2.VSVg (addgene#12260; addgene#8454) together with lentiCas9-Blast plasmid addgene#52962) was prepared and mixed with polyethylenimine (PEI) in a 1:6 ratio and transferred to the HEK-293 T cells. After 72 h, the medium was collected, centrifugated at 5000 rpm for 5 min, and filtered with a 0,45 µm poro syringe filter (Cultek, #15431231). PC3 cells were transduced with Lenti-Cas9 expressing lentivirus to induce the expression of Cas9 in presence of 8 µg/ml of polybrene. 48 h later, blasticidin selection (10 µg/ml) was performed for 5 days. Individual clones were expanded and the Cas9 presence was verified by Western-blot using Cas9 anti-mouse IgG and proliferation/migration test were performed to ensure no clonal effect was present (data not shown). Specific *MAP4K4* gRNAs were annealed and cloned in plasmid lentiGuide-puro plasmid (Addgene #52963) using BsmBI cloning site (Forward seq 5′-*CACCG*AGTTGGTCATCATGTCCTGG-3′ and reverse *AAAC*CCAGGACATGATGACCAACTC). Sanger sequencing confirmed the gRNA insertion and plasmid sequence. Viral productions to deliver gRNA in cells was performed as above using packaging and enveloping plasmids psPAX2 and pMD2.VSVg (addgene#12260; addgene#8454) together with plenti-Guide-puro (Addgene#52963) containing gRNAs or NTC sequence (Forward 5′-*CACCG*CGGCTGAGGCACCTGGTTTA-3′ and reverse *AAAC* TAAACCAGGTGCCTCAGCCGC-3′). PC3-Cas9 were transduced with lentivirus (MAP4K4-1) in presence of polybrene (8 µg/ml) to knock down the expression of *MAP4K4*. After 2 days, puromycin selection (1 µg/ µl) was added for 6 days. Clones were subcloned in a 96 multiwell plate and DNA was extracted using the Proteinase K method. Targeted region was PCR amplified, and sequence for the detection of indels. Presence of indels was used as inclusion criteria for HGK protein expression analysis assessed by Western-blot using HGK antibody followed by a secondary antibody conjugated to HRP.

### Migration assays

Wound healing-like migration was assessed by scratch assay using cell plates with a confluence of 100%. The cells were serum-deprived for 6 h before scratch was made with a pipette tip. Subsequently, they were kept in culture medium without serum and photographs were taken at 24 h with a phase contrast microscope, which were processed using the TScratch software.

For evaluation of migration through a transwell, the upper chamber with a 8 µm filter was filled with medium 0% FBS containing 25 000 cells, while the lower chamber was filled with 5% FBS medium or TNF-α (20 ng/ml). After 18 h, migration of the cells through the membrane was examined by fixing with paraformaldehyde 4% for 20 min and staining with crystal violet in aqueous solution (0.2%). Then, extra stain was removed, and cells were visualized with Eclipse TE300 Nikon microscope and photographed with digital DS-U2 camera. ImageJ was used to count the cells that traversed the membrane.

### Cell cycle analysis

Cells maintained in the indicated conditions were trypsinized and centrifuged at 2500 rpm 5 min at 4 °C, fixed with cold ethanol (70%) and washed with PBS. Cells resuspended in PBS were incubated 30 min with RNase (0.1 μg/ml) at 37 °C. Then, propidium iodide (0.05 μg/μl) was added and cell cycle analysed by flow cytometry. Modfit software was used to determine the percentage of cells in the different phases.

### Anchorage-dependent and -independent growth assays

Anchorage-dependent growth was evaluated by seeding 300 cells per 6 multi-well plate with RPMI/F12 10% FBS. After 8 days, the colonies were fixed and stained with crystal violet (0.2%, in 2% ethanol), counting the number of colonies with the ImageJ software. Anchorage independent growth was evaluated by preparing 24 multiwell plates with 0,5 ml of a mixture 1:1 of agar 1% and RPMI/F12 10% FBS. 3000 cells were seeded per 24 multiwell with RPMI/F12 10% FBS mixed 1:1 with agar 0,7%. Each sample was analysed per triplicate. Number of colonies were counted manually.

### Sprouting assay

Multicellular spheroids for the sprouting assay were initiated by seeding 625 PC3-NTC cell or HGK deleted PC3-HGK-KO-1 or PC3-HGK-KO-2 in several 25 µl hanging drops with RPMI/F12 medium. Under these conditions, cells aggregate into a single spheroid by 3–4 days. Spheroids were then transferred to 96 well plates which had been previously covered by a previously solidified layer of Matrigel and RPMI/F12 medium 1:1. Spheroids were also resuspended into a gelling solution comprised of Matrigel and RPMI/F12 Medium 1:1, which was then allowed to polymerize at 37 °C on the treated wells. Spheroids were feeded every 2–3 days. After 4–7 days of incubation, images of the embedded spheroids were taken under an inverted microscope. The numbers and lengths of capillary-like sprouts growing from each spheroid were measured using the software ImageJ (https://imagej.nih.gov/ij/).

### Proliferation and viability assays

Cell proliferation was assessed by cell counting manually using a hemocytometer. Briefly, cells were seeded onto 24-well plates (5 × 104 cells/well in 1000 ml of DMEM supplemented with 10% FBS). After 24, 48, or 72 h, cells were collected by trypsinization and counted using an inverted microscope. Non-viable cells were discriminated by trypan blue positive staining (0.1% Trypan Blue solution Sigma-Aldrich). Number of cells was normalized by initial number of total cells seeded. Cell viability was assessed by the MTS assay (CellTiter 96® Aqueous One solution cell proliferation assay, Promega). For the MTS assay, cells were seeded onto 96-well plates (1 × 104 cells/well in 100 μl of DMEM supplemented with 10% FBS). MTS was added after 24, 48, or 72 h, and absorbance was measured at 490 nm.

### Gene silencing by siRNA

siRNAs were obtained from Dharmacon (siMAP4K4 ON-TARGETplus SAMRT POOL L-003971–00-0005). Non-targeting control siRNAs were obtained from Dharmacon (D-001810–01-20). 5 × 10^4^ were initially transfected using Lipo-RNAiMAX with 25 nM of siRNA in suspension. After 24 h of incubation, adherent cells were resuspended again and re-transfected with 25 nM siRNA in suspension using Lipo-RNAiMAX following the instructions provided for the manufactor.

### Immunofluorescence staining

Cells were maintained on cover-glass objects coated with gelatine (0.2%) to 37 °C. They were fixed with paraformaldehyde (4%) for 20 min and permeabilized afterwards with Triton × 100 (0.3% PBS). Non-specific interactions were blocked by incubating cells 1 h in a BSA solution (1% in PBS). Subsequently, the cells were incubated in darkness with phalloidin conjugated with rhodamine-123 (1:500) (Sigma, P1951), vinculin (1:50) (CST#4650), and DAPI (1 µg/ml, Panreac, A4099) at 0.1% BSA-PBS for 1 h. Goat anti-rabbit Alexa Fluor 488 (Invitrogen A32731) was used as fluorescent secondary antibody. To visualize cells a fluorescence microscope (Eclipse TE300 Nikon) coupled to a digital camera DS-U2 was used. Specifically for supplementary Fig. 5S images were taken in Universidad Complutense de Madrid CAI Centro de Citometría y Microscopía de Fluorescencia using a LSCM Leica SP8 microscope.

### Survival analysis using mRNA expression data

Cox proportional hazards regression analysis was performed using the RNA-seq determiend gene expression values for MAP4K4. In the Cox regression we checked all available cutoff values between the lower and upper quartile and selected the best cutoff for the Kaplan–Meier plots. We computed False Disovery Rate (FDR) via the Benjamini–Hochberg method to correct for multiple hypothesis testing. Statistical significance was determined as FDR < 10%.

### Statistical analysis

The results are expressed as mean value ± S.E.M of 2–5 independent experiments or mean ± S.E. Statistical analyses were made by ordinary one-way ANOVA or two-way ANOVA multiple comparisons test depending on the experiments (*p* value ≤ 0.05 being considered significant). The GraphPadPrism 8.1 program (GraphPadforScience, San Diego, USA) has been used for representing all the results.

## Consent for publication

Authors declare their consent for publication.

## Results

### HGK is upregulated in metastatic prostate cancer cell lines

**Figure 1:**
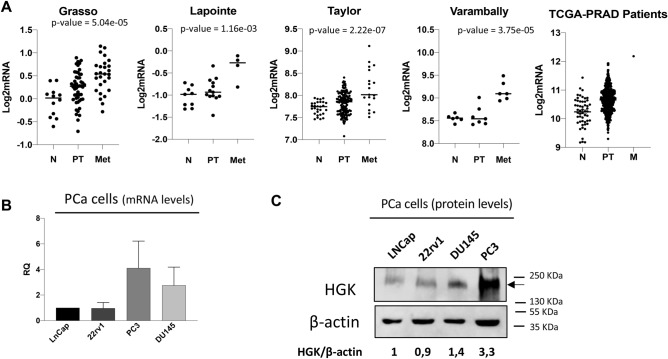
HGK is upregulated in PCa patient samples and cell lines. (**A**) *MAP4K4* mRNA levels in PCa patients and normal tissue obtained from indicated databases. (**B**) *MAP4K4* mRNA levels in the indicated PCa cell lines. (**C**) Western-blot analysis of HGK protein levels in the indicated PCa cell lines normalized with β-actin. Densitometric quantification of HGK/tubulin is shown. Full-length blots are presented in Supplementary Figure 2.

In order to shed some light on the role of HGK in metastasis and dissemination we decided to investigate its function on the invasive and migratory properties of PCa. First, we obtained publicly available RNA-seq data from 537 prostate adenocarcinoma (PRAD) patients from The Genome Cancer Atlas (TCGA) database. We compared *MAP4K4* mRNA expression levels between normal and tumoral samples and between primary tumor and metastatic samples and showed that *MAP4K4* levels were upregulated in the metastatic PCa sample as compared to primary tumor and normal tissue. TCGA data results were further supported by other four independent datasets(Grasso, Lapointe, Taylor and Varambally) (Fig. 1A). Our data base analysis suggested a positive correlation between *MAP4K4* expression and PCa dissemination. To validate these results and establish the importance of *MAP4K4* function in PCa cell motility, we analyzed the expression of *MAP4K4* in 4 different metastatic prostate cancer cell lines, LNCaP, 22rv1, PC3 and DU145. As shown in Fig. 1B, *MAP4K4* mRNA levels were upregulated in the highly metastatic cell lines, PC3 and DU145, compared to the low metastatic cell lines LNCaP and 22rv1. Thus, we analyzed HGK protein levels in the same cell lines and observed that HGK protein levels were higher in the highly metastatic PCa cells compared to low invasive cells (Fig. 1C), being PC3 cells those with a higher expression.

### HGK depletion or inhibition decreases tumor cell migration and adhesion

**Figure 2:**
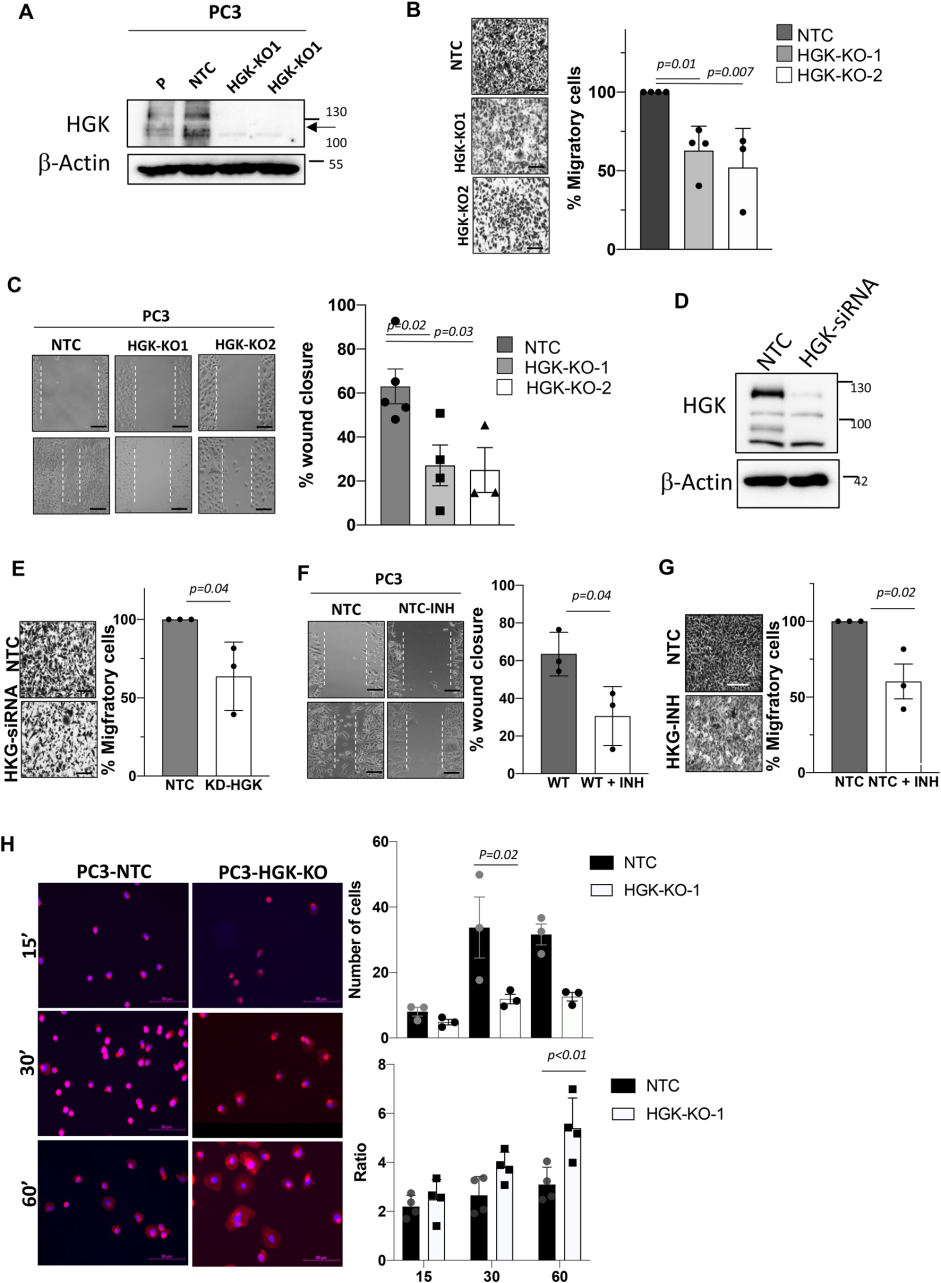
HGK down-regulation diminishes migration, while enhances adhesion in PCa cells. (**A**) Western-blot analysis of HGK normalized with β-actin in PC3 cells to confirm depletion. (**B**) Migration analysis in PC3 cells using FBS as a chemoattractant. Left panels, representative images of cells (bars: 100 µm); right panels, histograms showing the mean value ± S.E.M. of the number of migrating cells (n = 3). (**C**) Wound healing assay. Left panels, representative pictures of PC3 cells at time 0 and 24 h (bars: 100 µm); right panels, histograms showing the mean value ± S.E.M. of the percentage of wound closure (n = 3). (**D**) Western-blot analysis of HGK normalized with β-actin to confirm depletion after siRNA treatment in PC3 cells. (**E**) Migration analysis in HGK siRNA PC3 cells using FBS as chemoattractant. Left panels, representative images of migrating cells; (bars: 100 µm); right panels, histograms showing the mean value ± S.E.M. of the number of migrating cells (n = 3). (**F**) Wound assay analysis of untreated or HGK inhibitor treated PC3 cells. Left panels, representative pictures of cells at time 0 and 24 h (bars: 100 µm); right panels, histograms showing the mean value ± S.E.M. of percentage of wound closure (n = 3). (**G**) Migration analysis in PC3 cells treated with the HGK inhibitor using FBS as chemoattractant. Left panels, representative images of migrating cells (bars: 100 µm); right panels, histograms showing the mean value ± S.E.M. of the number of migrating cells (n = 3). (**H**) Adhesion analysis at 15, 30 or 60 min in the indicated PC3 cells. Left panels, representative images of adhered cells (bars: 50 µm). Right panels top, histograms showing the mean value ± S.E.M. of the percentage of adhered cells referred to non-silenced cells at 15 min (n = 3). Right panels bottom, histograms showing the mean value ± S.E.M. of the percentage of adhered cells or the ratio cytosol/nucleus referred to untreated cells at the indicated times (n = 3). Full-length blots are presented in Supplementary Figure 3.

To assess whether HGK upregulation was involved in the invasive properties of PCa cells, we decided to deplete HGK in the highly metastatic cell line PC3 using CRISPR approaches. HGK protein expression was efficiently depleted in two clones (HGK-KO-1 and 2) (Fig. 2A and supplementary Figure 1A and 1B), while non-targeting control (NTC) sgRNA had no effect on HGK levels. First, we evaluated the effect of HKG depletion in PCa cell lines on migration and adhesion. For migration characterization, we used transwell assays and found that migration was reduced in HGK-KO-1 and -2 cells as compared to NTC control cells, in response to FBS, used as chemoattractant (Fig. 2B). To ensure that the impaired migratory phenotype was not dependent on the transwell assay, we performed wound healing assays with the same cellular model. In agreement with our previous results, the motility and ability to close the wound of HGK depleted cells was reduced compared to NTC control cells (Fig. 2C). Additionally, to avoid proliferation interferences, wound closure assay after mitomycin treatment was performed, showing similar results in HGK depleted cells (Supplementary Figure 2).

To prove that the impaired migration phenotype was not a consequence of the genetic edition, we performed two different approaches, transient HGK silencing in PCa cells using specific siRNAs and inhibition with a pharmacological inhibitor (PF 06260933 dihydrochloride). As expected, after downregulating HGK expression (Fig. 2D), PC3 migration decreased (Fig. 2E). HGK protein expression was efficiently downregulated, while the non-targeting control siRNA had no effect (Fig. 2D). HGK chemical inhibition using HGK inhibitor and its corresponding control further validated our migration and wound healing impairment experiments (Fig. 2F, G), even in cells treated with mitomycin (supplementary Figure 2).

Since HGK regulates cellular functions that control cell adhesion, a process highly connected with the migratory process, we decided to analyze the impact of HGK depletion on the adhesive properties of PCa cells. As shown in Fig. 2H, the number of adhered cells was decreased in cells with HGK down-regulation compared to NTC cells (Fig. 2H, right panel top). Interestingly, we observed that cell morphology was different, so we decided, not only to analyze cell adhesion, but also evaluate the cell spreading. To assess the extent and dynamics of these processes, we analyzed the ratio of actin cytoskeleton vs nuclei area by comparing Phalloidin/DAPI staining of seeded cells on gelatin-coated coverslips. As shown in Fig. 2H, the spread ratio increases dramatically 1 h after cell seeding in HGK depleted cells, suggesting that their adhesion required more time. However, the spreading process is improved along the time, leading to a more stable adhesion, which might impede migration. Thus, loss of HGK function drives a transition of PCa cells towards a non-migratory state. Taken together, these findings indicate that HGK inhibition reduces the migratory capacity of PCa cells.

### HGK depletion alters tumorigenic properties of prostate cancer cells

**Figure 3:**
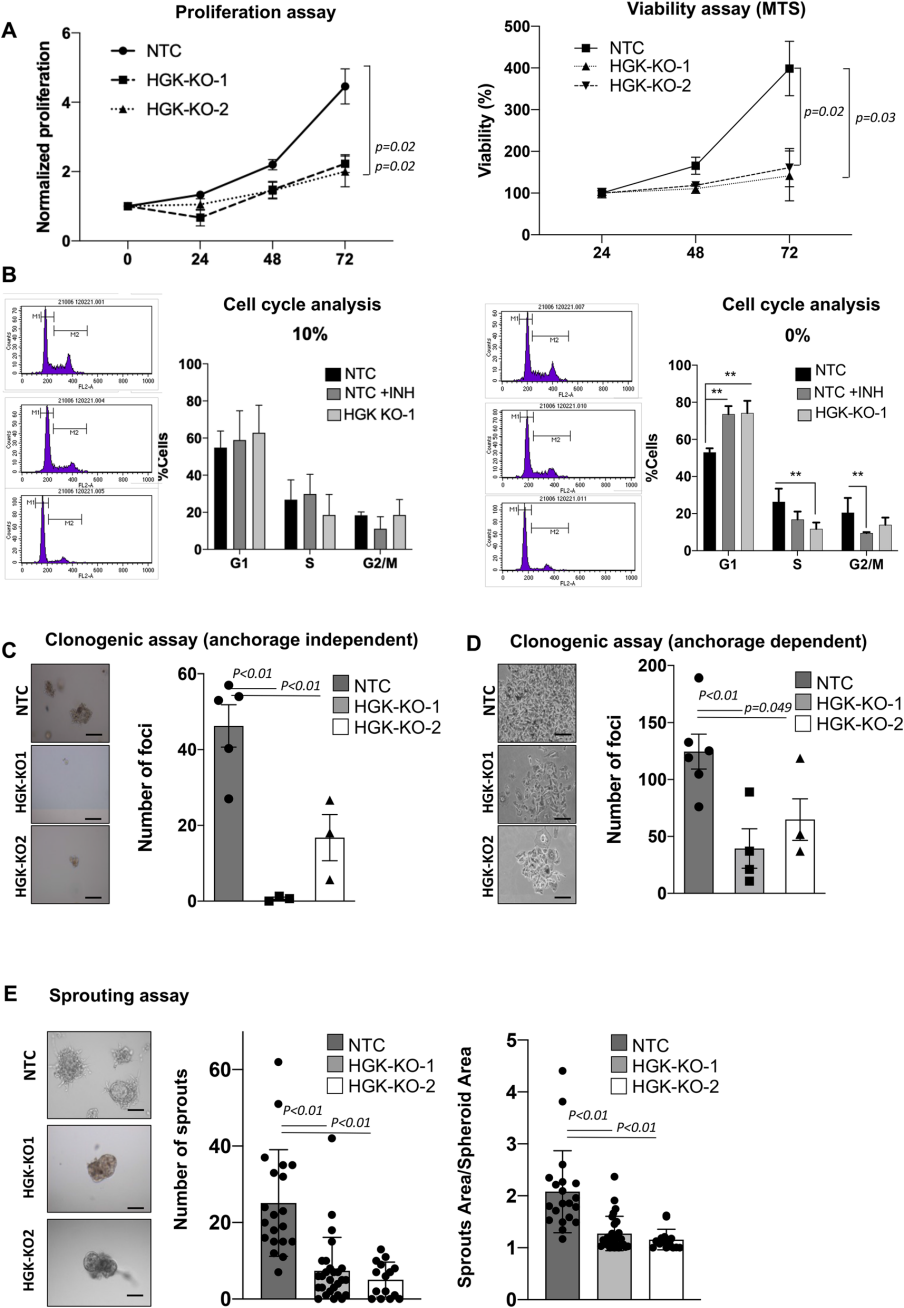
Effect of HGK deletion on tumorigenic and proliferative properties of PCa cells. (**A**) Proliferation analysis of NTC or HGK depleted PC3 cells. Left panels, normalized number of proliferative cells. Right panel, cell viability evaluated by MTS. (**B**) Cell cycle analysis: Cells were maintained in presence or absence of 10% FBS for 24 h and analyzed by flow cytometry using ModFit software. Left panel, representative images of the distribution of cell cycle phases; right panel, histogram showing the % of cells in the different phases of cell cycle ± S.E.M. (n = 3). (**C**) Anchorage-independent growth assay in PC3 cells. Left panel, representative images of a macroscopic view of foci (bars: 100 µm); right panel, histogram showing the mean value ± S.E.M. of the total foci number per field normalized to NTC (n = 3). (**D**) Anchorage-dependent growth assays in PC3 cells. Left panels, representative images of a macroscopic view of foci (bars: 100 µm); right panels, histograms showing the mean value ± S.E.M. of the foci number per field normalized to NTC (n = 3). (**E**) Sprouting analysis in NTC or HGK depleted PC3 cells. Left panels, representative images of sprouts formation; right panels, histograms showing the mean value ± S.E.M. of the number of sprouts per spheroid or the sprout area per spheroid area (n = 3). Bars: 50 µm.

HGK is known to mediate proliferation in T cells and a number of cancer cells, such as lung and gastric cancer^8,21,22^. Therefore, we decided to study the effect of deleting HGK on the growth and tumorigenic properties of PC3 cells by performing several functional studies. First, we found that cell proliferation and viability were decreased in HGK depleted cells after 48 or 72 h (Fig. 3A). A cell cycle analysis by flow cytometry showed no statistically significant differences between HGK depleted and non-depleted cells, when cells are maintained in the presence of serum. However, in serum deprived cells, HGK pharmacological inhibition and HGK depletion significatively decrease S and G2/M phases leading to the accumulation of cells in G1 (Fig. 3B). In agreement with this result, anchorage-dependent and –independent growth assays revealed a decreased number of foci upon HGK depletion in PCa cells as compared to NTC cells (Fig. 3C,D).

The migration of human cancer cells is highly dependent on extracellular matrix and stromal cellular constituents, supported by engagement of different molecular machinery of migration in 2D versus 3D tissue architectures. We sought to determine the efficacy of HGK inhibition using the sprouting assay to assess 3D tumor growth and scattering of PCa cells. PC3-NTC cells manifest enhanced sprouting capacity compared to HGK depleted cells, as measured by the number of sprouts and the sprout length in the three-dimensional spheroid sprouting assay conducted in the presence of Matrigel matrix (Fig. 3E). Hence, our results suggest that HGK promotes PCa cells proliferation. Furthermore, it also induces PCa cells sprouting in 3E, suggesting HGK also promotes invasion in PCa cells.

### HGK regulates cell morphology and F-actin cytoskeletal organization

**Figure 4:**
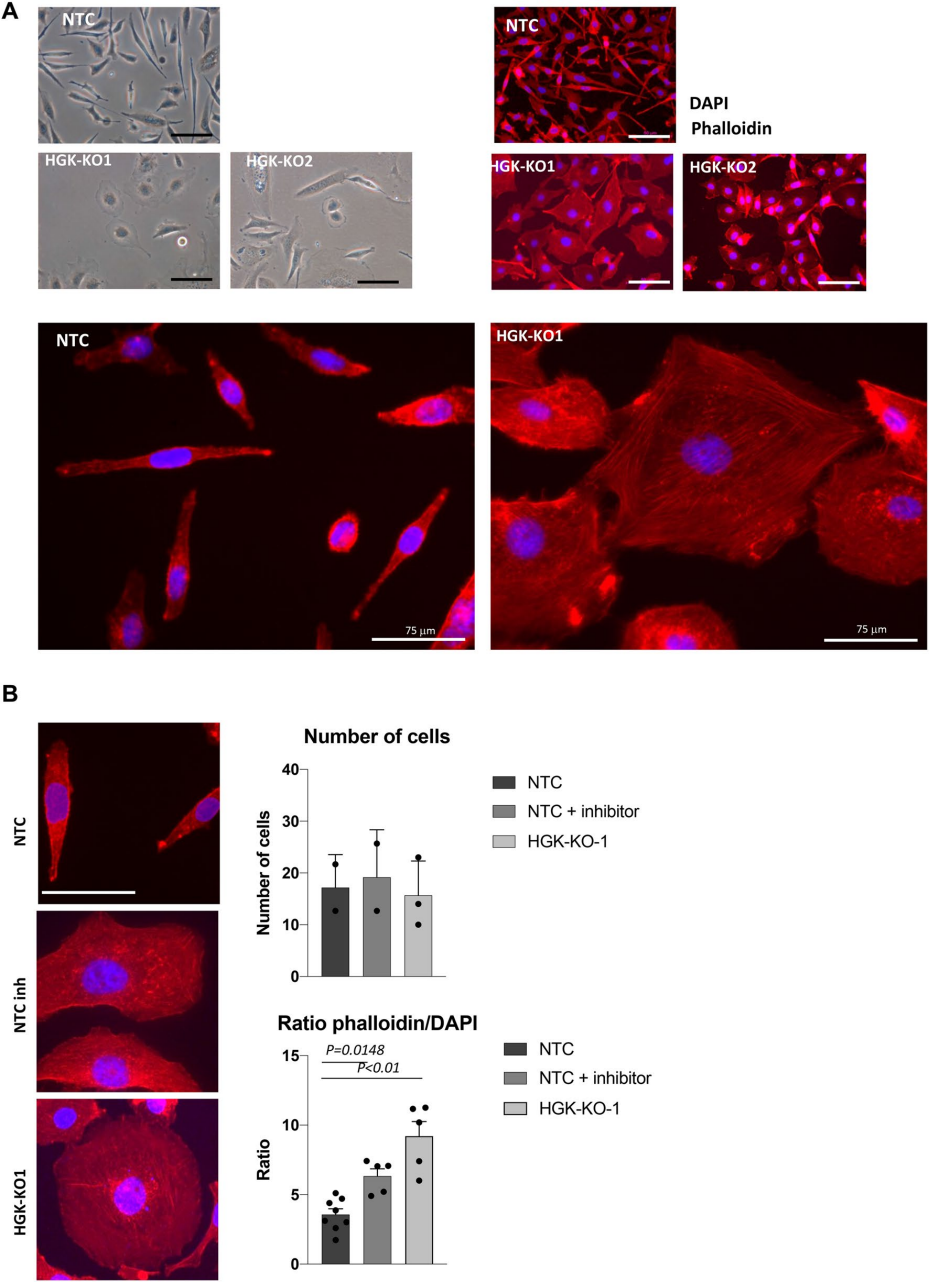
HGK depletion alters cell actin cytoskeleton organization. (**A**) Top left panel, Phase-contrast microscopy images of NTC and HGK depleted PC3 cells maintained in the presence of 10% FBS for 24 h. Top Right panel, Immuno-fluorescence microscopy images of phalloidin staining (*red*) in NTC and HGK depleted PC3 cells. Cell nuclei were stained with DAPI (*blue*). Scale bars: 75 μm. An amplification of images is depicted. (**B**) Adhesion analysis at 24 h in indicated cells. Left panels, representative images of adhered PC3 cells. Right top panels, histograms showing the mean value ± S.E.M. of the percentage of adhered cells referred to non-silenced cells at 24 h (n = 3). Right bottom panel, histograms showing the mean value ± S.E.M. of the ratio cytosol/nucleus referred to untreated cells at 24 h.

Since our results have shown that HGK regulates migration and invasion, and these two processes are regulated by actin dynamics, we decided to analyze cell morphology of PCa cells. Figure 4A shows that HGK depleted cells switch from a spindle shaped invasive state to a cobblestone-like resting state. This altered morphology was also detected in anchorage-dependent growth assays. In order to characterize this difference in morphology, cell spreading was quantified by measuring cell area 24 h after being seeded. HGK depleted PC3 cells clearly increased their cell territories over time, whereas the average of NTC cell area remained unchanged. Interestingly, HGK pharmacological inhibition confirmed the results, so that treated cells had a higher mature spread ratio than untreated cells (Fig. 4B).

Since the cell morphology is directly influenced by the intracellular actin cytoskeleton, we study if HGK depletion affected F-actin organization. As shown in Fig. 4A, a closer analysis of HGK depleted cells exhibit some differences in their F-actin content and organization, compared to PC3-NTC. PC3-NTC showed a higher amount of parallel actin fibers, which dictates a fibroblast-like morphology. In contrast, HGK depleted cells contained more stress fibers and FA and less parallel actin fibers than PC3 NTC (Fig. 4A,B).

### HGK regulates ruffles formation, cell motility and dissemination of PCa cells in response to TNF‐α

**Figure 5:**
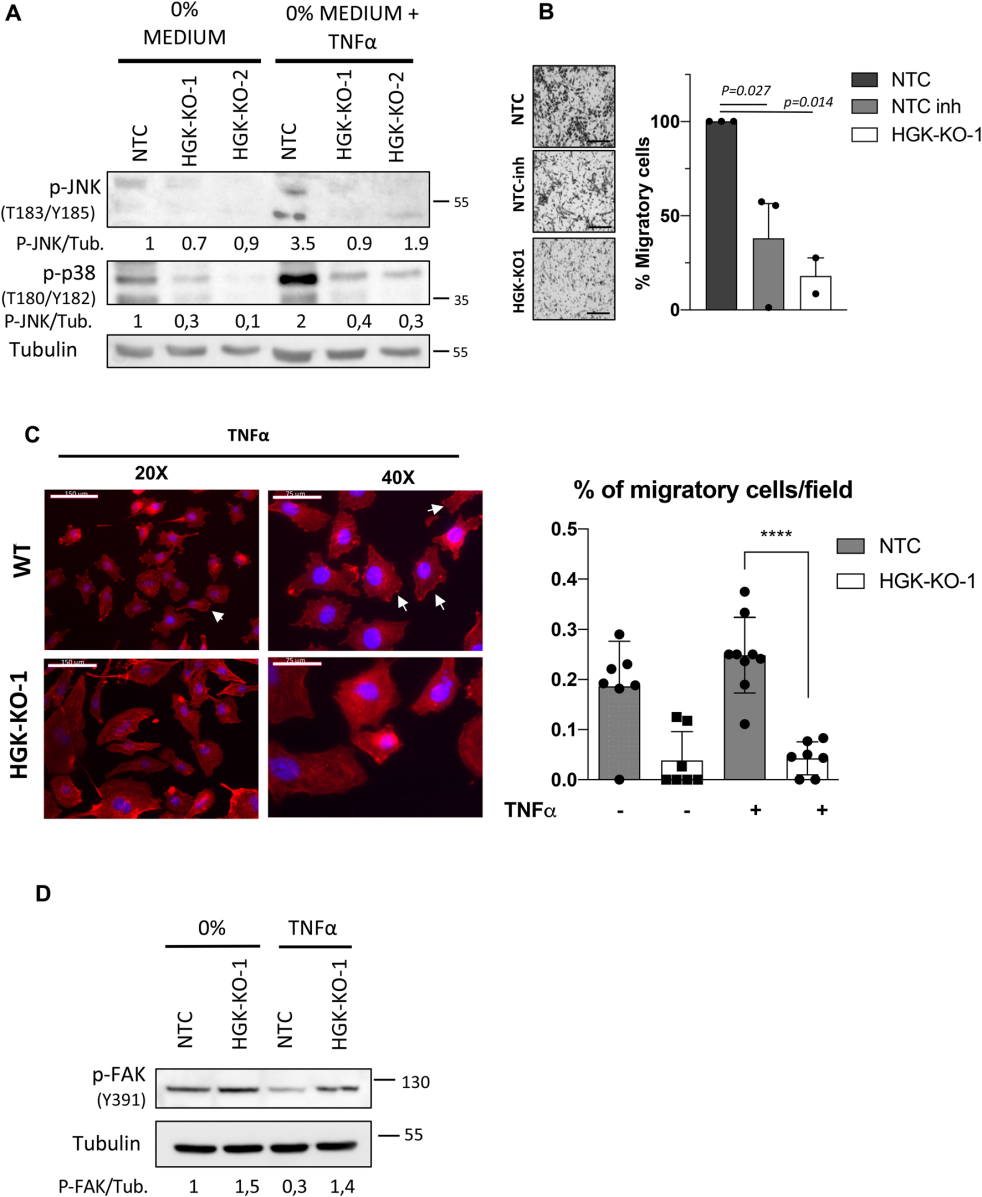
HGK regulates ruffle formation and cancer cell motility in response to TNF-α. (**A**) Representative Western-blot analysis of the phosphorylated levels of JNKs and p38 proteins normalized with β-actin on the indicated PC3 cells. Serum-starved cells (for 16 h) were stimulated with TNF-α for 15 min or maintained untreated. Densitometric quantification are shown (**B**) Migration analysis of the indicated PC3 cell using TNF-α as chemoattractant. Left panels, representative images of migrating cells (bars: 100 µm); right panels, histograms showing the mean value ± S.E.M. of the number of migrating cells (n = 3). (**C**) Immuno-fluorescence microscopy images of phalloidin staining (*red*) in NTC and HGK depleted PC3 cells. Serum starved cells were stimulated with TNF-α for 15 min. Cell nuclei were stained with DAPI (*blue*). Scale bars: 150 and 75 μm. Right panel, histograms showing the mean value ± S.E.M. of cells with ruffles referred to non-silenced cells (n = 3). (**D**) Representative western-blot analysis of phosphorylated levels of JNKs and FAK-Y397 normalized with β-actin. Serum-starved PC3 cells (for 16 h) were stimulated with TNF-α for 15 min or maintained untreated. Full-length blots are presented in Supplementary Figure 3.

The identification of downstream signaling mediators of HGK is crucial for understanding the mechanisms underlying HGK regulation in PCa metastasis to the bone. The bone marrow is a common and ideal destination for metastases. It contains sinusoidal vasculature, which is beneficial for circulating cancer cell migration. Bone marrow turnover and activity involves the production of numerous cytokines that co-incidentally can also attract and support metastatic PCa cells^23^. TNF‐α is a cytokine commonly found in bone marrow and one of the main known activators of HGK^24^. It is also a strong mediator for PCa development and generation of metastasis. To test the role of HGK in TNF-α signaling we compared the activation status of JNK1/2 and p38 MAPKs in PCa NTC or HGK depleted cells in response to TNF‐α (Fig. 5A). As expected, depleted cells failed to activate JNKs and p38 MAPKs in response to TNF‐α (Fig. 5A), while no differences were detected in the activation of ERKs (supplementary Fig. 3). These results suggest that HGK might mediate TNF-α effects through regulation of JNK1/2 and p38 MAPKs in prostate cancer metastasis. We also analyzed the migratory properties of PCa using TNF‐α as a chemoattractant stimulus and found that migration was strongly reduced in depleted cells as compared to NTC control cells (Fig. 5B). In agreement with this result, the treatment with HGK inhibitor displayed a similar effect than its genetic deletion (Fig. 5B). Furthermore, TNF-α induces a substantial reorganization of actin cytoskeleton and focal adhesions in epithelial cells^25^ and strongly mediates tumor dissemination in PCa cells^26^. Thus, we decided to analyze if HGK was involved in the actin cytoeskeleton reorganization in response to TNF‐α, facilitating cell motility. We observed that TNF‐α stimulation induced a proper formation of dorsal arcs and ventral stress fibers with a strong cortical ring, characteristic of migratory cells. In contrast, KO cells organization was completely different with absence of a cortical ring and presence of a perinuclear actin cap (Fig. 5C and supplementary figure 5).

In order to investigate the molecular mechanism by which HGK regulates the actin cytoskeleton and FA, we analyzed the phosphorylated status of one of the main regulators, the focal adhesion kinase (FAK). FAK is a crucial signaling component that is activated by numerous stimuli and functions as an integrator that controls cell motility. It exerts its activity by influencing the cytoskeleton, the structure of cell adhesion sites, as well as membrane protrusions. FAK autophosphorylation on Y397 residue has been linked to maximum adhesion levels, and it is lost upon cell detachment. Additionally, Y397 phosphorylation is essential for development of mature FA. The distribution of vinculin, a main component of the mature focal adhesion complex, was also altered in HGK depleted cells. HGK-KO cells displayed a cytoplasmic dotted distribution of vinculin, usually associated with mature FAs, while in NTC cells vinculin expression was more disperse and concentrated in proximity to the nucleus (Supplementary figure 4). Figure 5D shows that depleted cells display a higher level of FAK-Y397 than NTC cells under basal condition and in response to TNF‐α stimulation. Interestingly, Y397 phosphorylation drops in NTC cells in response to TNF-α, probably favoring FA turnover, cell spreading and migration, while HGK depleted cells fail to response to TNF-α, leading to an aberrant regulation of FA dynamics, reduced migration and invasion.

### High levels of HGK predict a poor prognosis in PCa patients

**Figure 6:**
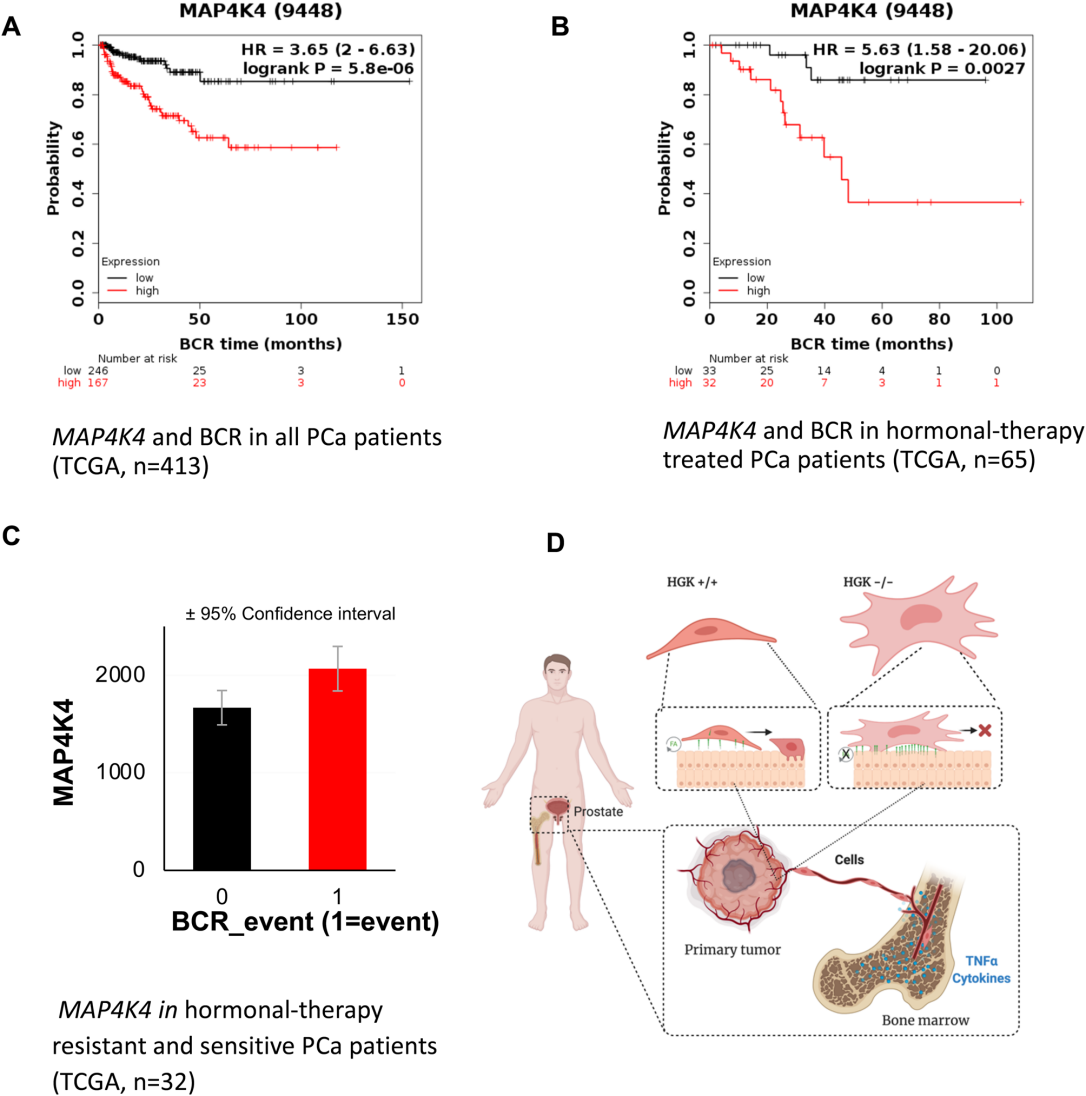
High levels of HGK predict a poor prognosis in PCa patients. (**A**) Kaplan–Meier curves showing the difference in BCR-free survival between patients with high and low expression levels of *MAP4K4*. (**B**) Kaplan–Meier curves showing the difference in BCR-free survival between hormonal-therapy treated patients with high and low expression levels of *MAP4K*. (**C**) *MAP4K4* expression in hormonal-therapy resistant and sensitive PCa patients (TCGA, cutoff for resistance: relapse before 40 months, n = 32). (**D**) Schematic summarizing of the regulation of Focal Adhesion (FA) turnover and its impact on cellular motility by HGK.

To further explore the role of this kinase in the metastatic process of prostate cancer in patients, we analyzed the association of HGK with cancer relapse. High expression of the tumor biomarker PSA measured in blood samples is usually the first sign of tumor relapse, a surrogative marker for metastatic spreading. Kaplan–Meier method was used to compare biochemical recurrence (BCR)-free survival of patients with high and low *MAP4K4* expression as described in material and methods. *MAP4K4* mRNA expression across all PCa tissues was used to classify all cases into high (n = 167) and low HGK expression (n = 246). Figure 6A show that patients with high expression of HGK show shorter BCR-free survival (HR 3.65, CI 2–6.63, *p* = 5.8e−06) compared with those with low expression. Additionally, we analyzed the BCR-free survival in hormone resistant patients. These patients have developed resistance to hormone therapy, a hallmark of the evolution to castration resistant prostate cancer. Figure 6B shows that hormonal-treated PCa patients with higher expression of HGK showed shorter BCR-free survival (HR 5.63, CI 1.58–20.06, *p* = 0.0027) compared to those with lower expression. We also analyzed the expression levels of *MAP4K4* in hormone resistant patients. As it can be seen in Fig. 6C, and consistent with our previous results, *MAP4K4* expression was higher in hormonal therapy resistant PCa patients than sensitive.

## Discussion

Several efforts have been made to characterize PCa tumor development. Correspondingly, there is a more comprehensible idea of the genes and signaling beneath this process than the understanding of the PCa metastatic process. Consequently, there are several therapeutic strategies for PCa tumors that remain effective in less aggressive states and as a result the disease in patients diagnosed early has a good prognosis. However, the picture is completely different for metastatic PCa. There is still no effective treatment for this stage of the disease and hence, there is an urgent need to find key gene regulators of this process, to be used as both, prognosis biomarkers and more importantly, therapeutic targets that could improve metastatic PCa survival rate. Our findings point out to HGK, a MAPK family member, as a critical regulator of PCa tumor progression and its implication in regulating focal cell adhesion signaling.

Our results showed that HGK levels were lower in benign prostate cells than in PCa cells. In fact, the hormone-independent and highly invasive PC3 PCa cell line exhibited the highest HGK protein levels. These findings indicated that HGK upregulation correlates with human PCa stages. HGK has been widely associated with the development of epithelial cancers. Its upregulation was associated with poor patient prognosis in colorectal cancer^11^, hepatocellular carcinoma^10^, pancreatic ductal adenocarcinoma^12^, lung adenocarcinoma^13^ and prostate cancer^27^. In addition, HGK protein levels were elevated in lung adenocarcinoma^8^, pancreatic cancer^9^, prostate cancer^27^, brain and kidney tumors and glioblastoma^7^. Numerous laboratories underscored important roles for HGK in cell proliferation and invasion in other cellular models. For example, high levels of *MAP4K4* mRNA expression augmented proliferation and invasion in ovarian cancer^6,14^ and medulloblastoma^15^.

We found that a high *MAP4K4* expression correlates with an enhanced proliferation and tumorigenic abilities of PCa cells and its depletion strongly downregulates these processes in in vitro assays. Hence, HGK seems to play an important role in PCa tumor progression by promoting tumorigenesis and dissemination. In agreement with our results, there is a growing body of evidence for the involvement of HGK in cancer cell migration and invasion. Former screenings for positive modulators of tumor cellular motility identified HGK in glioblastoma^16^, ovarian cancer^6^ and prostate cancer cell lines^18^. Despite the several evidences of association, the specific underlying mechanisms through which HGK influences invasion are not yet understood. For example, Miao et al. proposes that HGK acts through an upregulation of *Myc* oncogene^18^. Collins et al. indicates that HGK signals through JNK in an ovarian carcinoma cell line^6^. Gao et al. points out to the upregulation of MAPKs in lung adenocarcinoma^8^ and Prolo et al. suggests that HGK promotes an EMT reprograming in glioblastoma^19^. Protein function often depends on tissue expression and/or tumor type, but the disparity of mechanisms proposed for HGK reflects a lack of knowledge of its biology. Interestingly, none of the previous work studied the relation between HGK and cytoskeleton regulation.

According to our data, HGK depletion induces a strong modification of the actin cytoskeleton, altering the shape and morphology, as well as the area influenced by the cell. This altered morphology and shape has also been observed in other studies with other cellular models after HGK depletion or inhibition^28^ and^15^. Our results agree with Yue et al. observations in his conditional HGK KO mouse model, which displays an impaired migration and altered morphology of non-tumor cells (keratinocytes) as a consequence of a disorganized actin cytoskeleton^17^. Additionally, Yue’s HGK mouse model showed that HGK promotes FA dynamics by regulating IQSEC1/Arf6 pathway, controlling internalization and endocytosis of integrin in endothelial cells^17^. FAs are assembled at ECM–integrin junctions linking cytoskeletal and signaling proteins, such as Abl, Src, and Focal Adhesion Kinase (FAK), controlling cell adhesion, spreading and migration^29^. We observed a similar effect to the one observed by Yue et al., either by genetic ablation or pharmacological inhibition of HGK in PCa. Our results showed that HGK, not only increases the ability of the cells to quickly adhere, but also decreases the cell spreading process and promotes migration in response to TNF-α and other cytokines most likely through actin cytoskeleton regulation. Several evidences suggest that HGK regulates cytoskeleton and cell adhesion to matrix via FA disassembly and FA–actin stability through Arf6 activation^30–33^. In addition, phosphorylation of ERM proteins and Arp2 provides another mechanism for HGK-induced actin filament nucleation and lamellipodium formation in response to EGFR activation^34^. More importantly, here we also show that HGK depletion reduces adhesion, while increasing cell spreading probably through FAs stabilization, impairing cell migration. As previously reported, FAK activity is essential to regulate the FA dynamics, characterized by continual bouts of formation, maturation and disassembly. FAK autophosphorylation on Y397 residue is essential for its association with Src and it has been linked to adhesion and FAs turnover^35^. FAK ability to promote both the maturation and turnover of focal contacts is related to its dual role as a signaling kinase and an adaptor/scaffold protein. This ability places FAK as a dual modulator, able to regulate not only adhesion, but also migration^36^. Our analysis show that HGK depleted cells display higher levels of the autophosphorylated Y397-FAK residue than NTC cells in response to TNF‐α stimulation, as well as an increase of the immunoreactivity to vinculin suggesting a higher number of mature FAs. This higher phosphorylation is observed also under basal condition. Interestingly, Y397 phosphorylation drops in TNF-α treated NTC cells favoring FA turnover by accelerating the disassembly of mature FA and promoting the formation of nascent FA. This process will favor cell migration in response to TNF-α. On the contrary, HGK depleted cells response to TNF-α leads to aberrant regulation of FA dynamics, resulting in a less migratory phenotype (Fig. 6D). Moreover, TNF-α increases the activation of different signaling pathways, including FAK phosphorylation in HGK depleted cells. This response indicates that HGK could regulate the phosphorylation of FAK indirectly. Additional signaling pathways could contribute to this effect such as the wnt pathway, a known mediator of PCa stemeness and metastasis^37^ that can be modulated by TNF‐α^38^. Hence, the disturbance of F-actin via GSK-3β inhibition leads to a decreased stemness and bone metastasis in prostate cancer^39^. Preliminary data (not shown) from our lab indicates that HGK depleted cells failed to activate ATF2 in response to WNT5A, although further analyses are needed to characterize the potential implication of HGK in this pathway.

Finally, we found that *MAP4K4* expression at both mRNA and protein levels were up-regulated in PCa cells at advanced pathological or clinical stages, being identified as an unfavorable prognostic factor of BCR-free survival in PCa. Furthermore, our results show that high *MAP4K4* expression is dramatically associated with aggressive tumor progression in patients with PCa, short overall survival time and short BCR-free survival time.

In conclusion, our data strongly suggest that HGK upregulation may play a pivotal role in the characteristics and aggressive behaviour of PCa, especially in groups with advanced pathological disease. Furthermore, our results showed that HGK increases the migratory capacity, loss of adherence and proliferation and survival abilities of the PCa cells analyzed. Loss of HGK function drives prostate cancer cells into a non-invasive state, suggesting that HGK is required for the maintenance of the malignant phenotype of metastatic prostate carcinoma. Additionally, our findings indicate that HGK upregulation is associated with poor prognosis in patients with PCa pointing to HGK as a new bad prognosis biomarker that could be also used to distinguish patients with aggressive PCa. Although further studies will be necessary to gain a full understanding of the underlying molecular mechanisms, we have shown that its pharmacological inhibition could be an effective therapeutic approach to target prostate carcinoma in combination with other interventions.

## Supplementary Information

Supplementary Information.

## Data Availability

All data is available upon request.
