# Supplementary material for: HGK promotes metastatic dissemination in prostate cancer
Source: Sci Rep. 2021 Jun 10;11:12287. doi: 10.1038/s41598-021-91292-2 (PMC8192525; doi:10.1038/s41598-021-91292-2)

HGK promotes metastatic dissemination in prostate cancer

Sara Garcia-Garcia[1+], Maria Rodrigo-Faus[1+], Noelia Fonseca[1], Sara Manzano[1,2], Balázs Győrffy[3], Alberto Ocaña[2], Paloma Bragado[1,2], Almudena Porras[1,2] and Alvaro Gutierrez-Uzquiza[1,2 *]

#1 Departamento de Bioquímica y Biología Molecular, Facultad de Farmacia, Universidad Complutense de Madrid; #2 Instituto de Investigación Sanitaria del Hospital Clínico San Carlos (IdISSC), Madrid, Spain. #3 Semmelweis University Dept. of Bioinformatics and 2nd Dept of Pediatrics, Budapest, Hungary and TTK Cancer Biomarker Research Group, Budapest, Hungary

+ equally contribution

* Correspondence: A. Gutierrez-Uzquiza, Departamento de Bioquímica y Biología Molecular, Facultad de Farmacia, UCM, Ciudad Universitaria; IdISSC, Madrid, Spain. Tel.: +34 913941854; e-mail: alguuz@ucm.es.

Supplemental information

The supplemental information contain **supplemental methods,** where a detailed information about methods used in supplementary figures are included. This section also contains uncropped images of the western blot results presented in this manuscript.

1-Supplementary methods:

RNA Isolation, Reverse Transcription-PCR, q-PCR analysis and Sanger sequencing were performed following the protocols indicated on material and methods section.

Wound healing-like migration was assessed by scratch assay using cell plates with a confluence of 100%. The cells were serum-deprived for 6 hours before scratch was made with a pipette tip. Subsequently, they were kept in culture medium without serum and photographs were taken at 24 hours with a phase contrast microscope, which were processed using the TScratch software.

Western blots were performed following the protocols indicated on material and methods section. PVDF membranes were incubated with the following primary antibodies 1:1000 diluted in 5% BSA TTBS overnight. Actin CST-#3700, P-ERKs CST-#9212 were used as primary antibodies.

Cells were maintained on cover-glass objects coated with gelatine (0,2%) to 37 º C. They were fixed with paraformaldehyde (4%) for 20 minutes and permeabilized afterwards with Triton x100 (0,3% PBS). Non-specific interactions were blocked by incubating cells 1h in a BSA solution (1% in PBS). Subsequently, the cells were incubated in darkness with phalloidin conjugated with rhodamine-123 (1:500) (Sigma, P1951), vinculin (1:50) (CST#4650), and DAPI (1 µg/ml, Panreac, A4099) at 0.1% BSA-PBS for 1h. Goat anti-rabbit Alexa Fluor 488 (Invitrogen A32731) was used as fluorescent secondary antibody. To visualize cells a fluorescence microscope (Eclipse TE300 Nikon) coupled to a digital camera DS-U2 and confocal Nikon S8 were used.

2-Supplementary figures: 9 figures with additional data and uncropped images.

Supplementary Figure 1: HGK is upregulated in metastatic PCa cell PC3 cell line

a

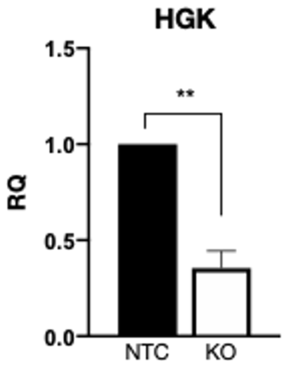

Supplementary Figure 1A: *MAP4K4* mRNA levels in PC3-Cas9 cells. *MAP4K4* mRNA levels are downregulated in PC3-Cas9 HGK-KO cells compared to PC3-Cas9 NTC. (n=3).

b

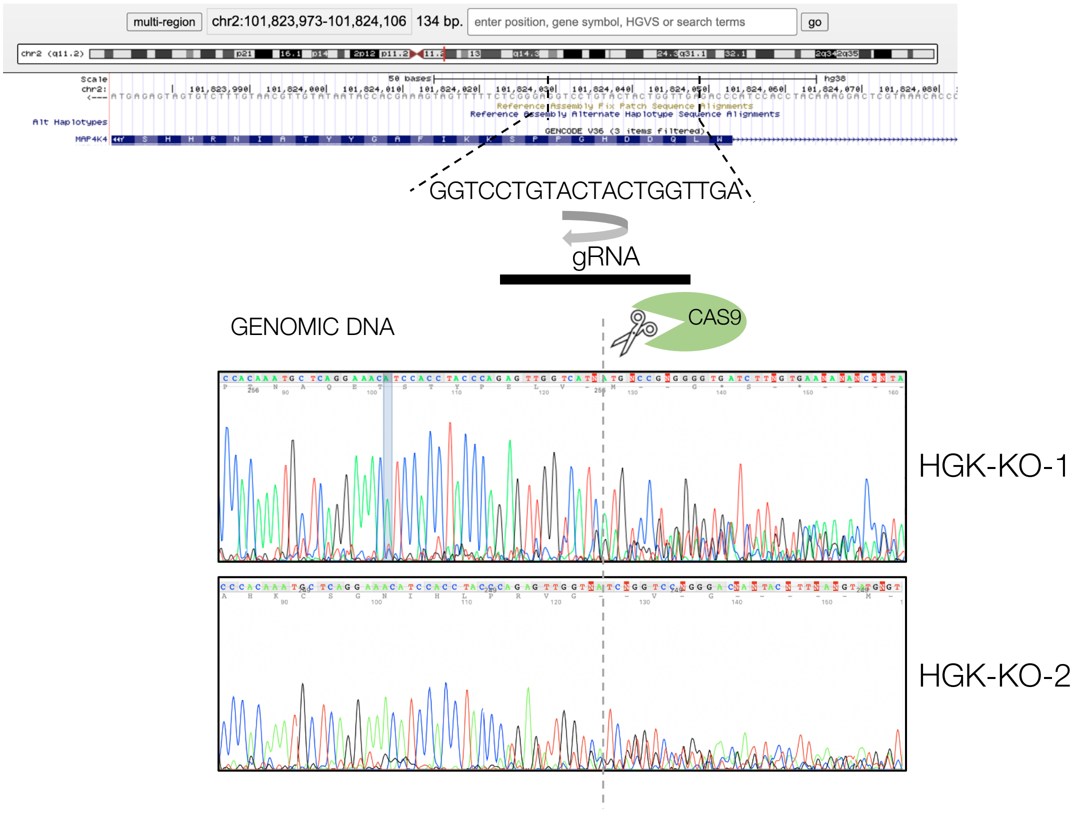

Supplementary Figure 1B: Knock-out of *MAP4K4* in PC3 cells. Schematic drawing of targeted genome editing at *MAP4K4* locus using CRISPR/Cas9. Sanger sequence traces of genomic DNA after the knock-out are shown.

Supplementary Figure 2: HGK down-regulation diminishes migration in PCa cells

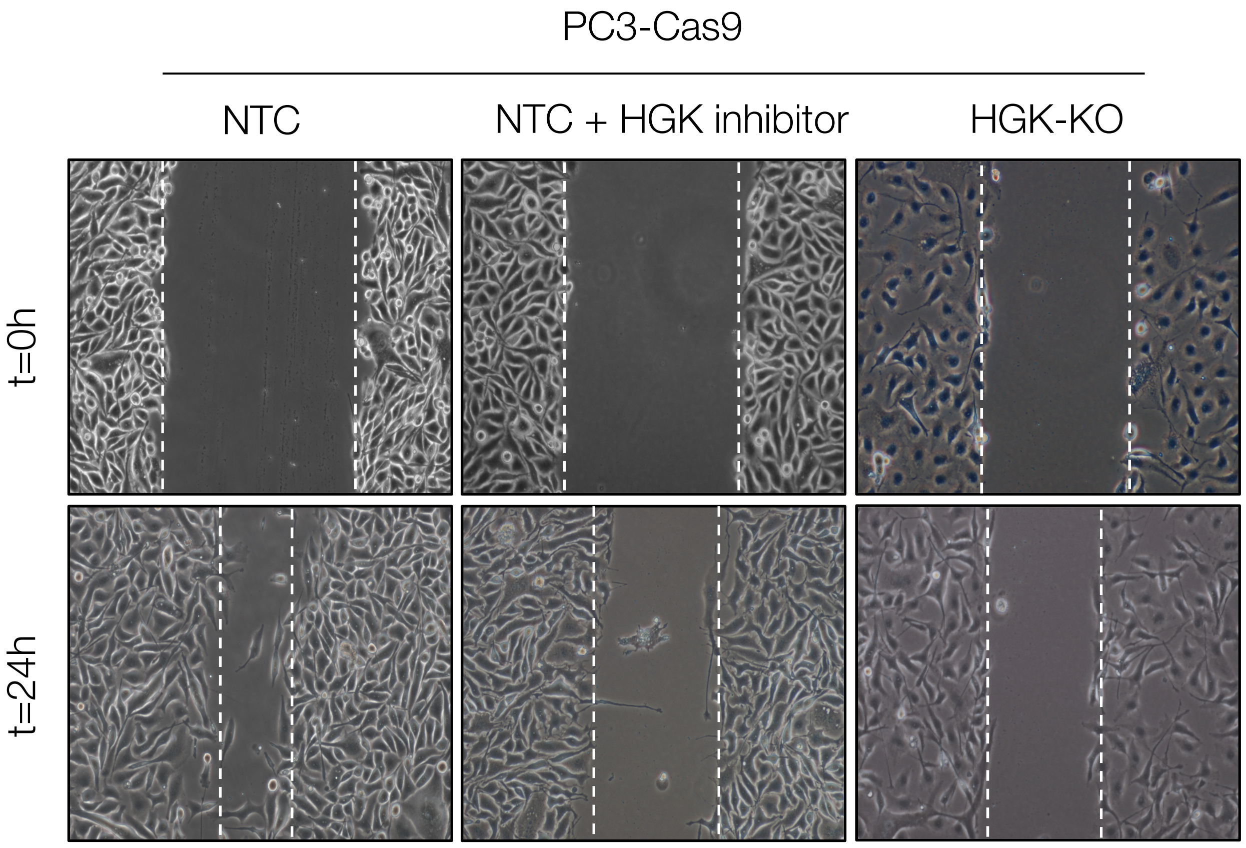

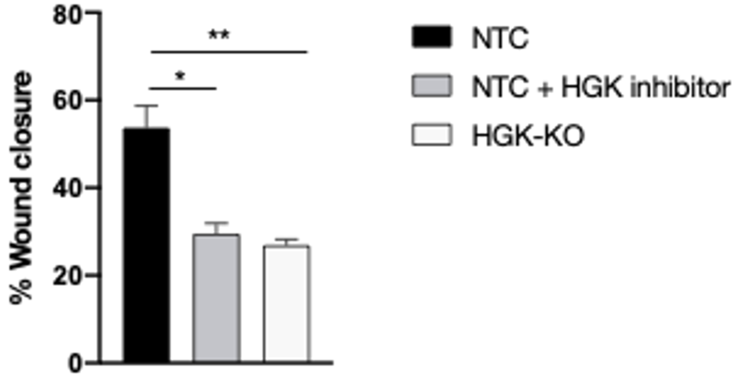

Supplementary Figure 2: HGK down-regulation diminishes migration in PCa cells. Wound assay analysis after mitomycin C pre-treatment of untreated, HGK inhibitor treated and KO PC3 cells. Top panel, representative pictures of cells at time 0 and 24h (bars: 100 µm); bottom panel, histograms showing the mean value ± S.E.M. of percentage of wound closure (n=3).

Supplementary Figure 3: HGK depletion does not affect ERK activation in response to TNF-α.

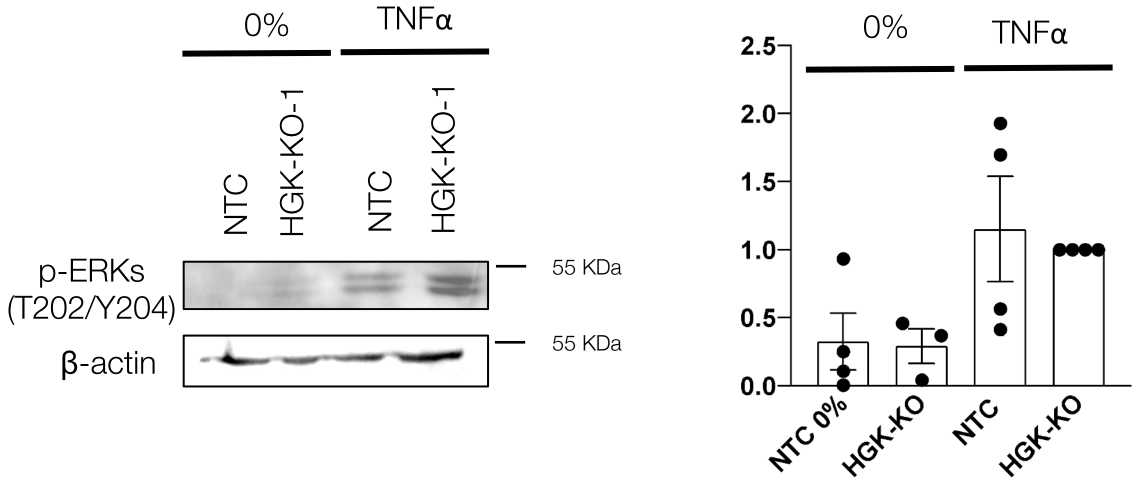

Supplementary Figure 3: HGK depletion does not affect ERK activation in response to TNF-α. Representative Western-blot analysis of the phosphorylated levels of ERKs proteins normalized with β-actin on the indicated PC3 cells. Serum-starved cells (for 16h) were stimulated with TNF-α for 15 min or maintained untreated. Densitometric quantification are shown.

Supplementary Figure 4: HGK is implicated in the regulation of focal adhesion assembly and disassembly.

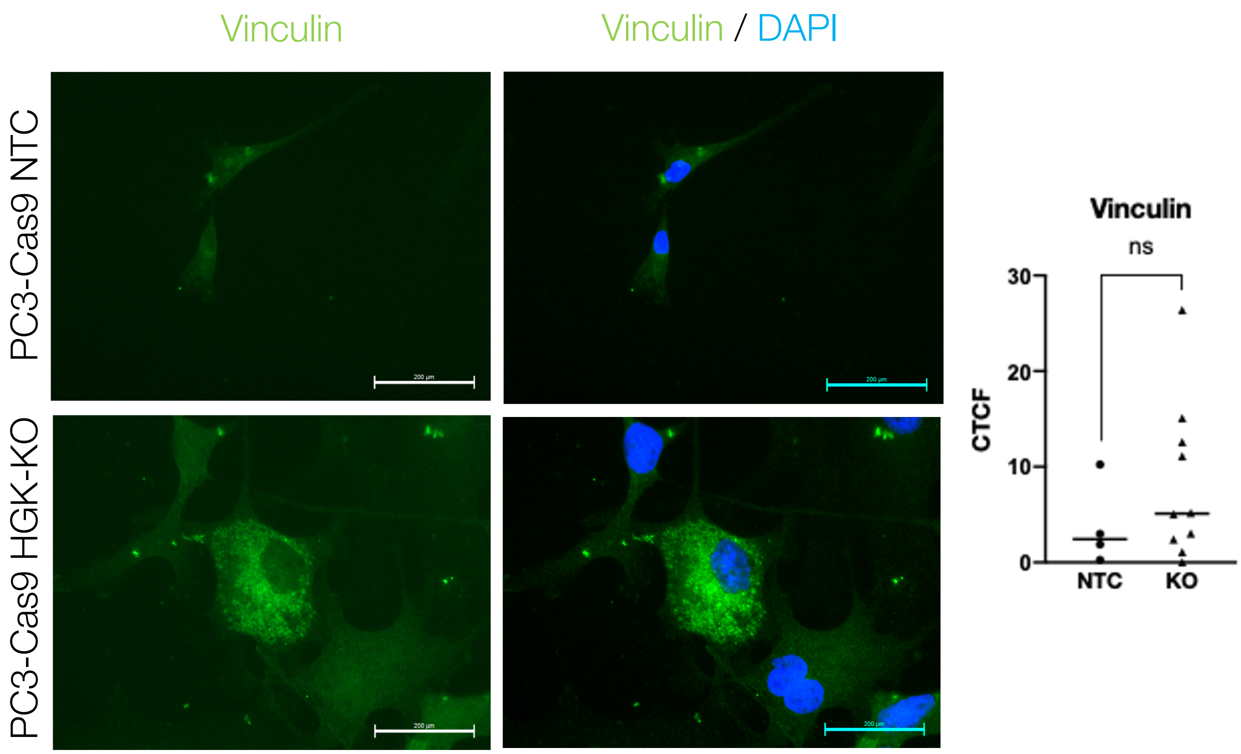

Supplementary Figure 4: HGK is implicated in the regulation of focal adhesion assembly and disassembly. Immuno-fluorescence microscopy images of vinculin (*green*) in NTC and HGK depleted PC3 cells. Cell nuclei were stained with DAPI (*blue*). Scale bars: 100 µm. Right graph represents the quantification of Vinculin CTCF (corrected total cell fluorescence).

Supplementary Figure 5: Pictures of migratory PC3 cells

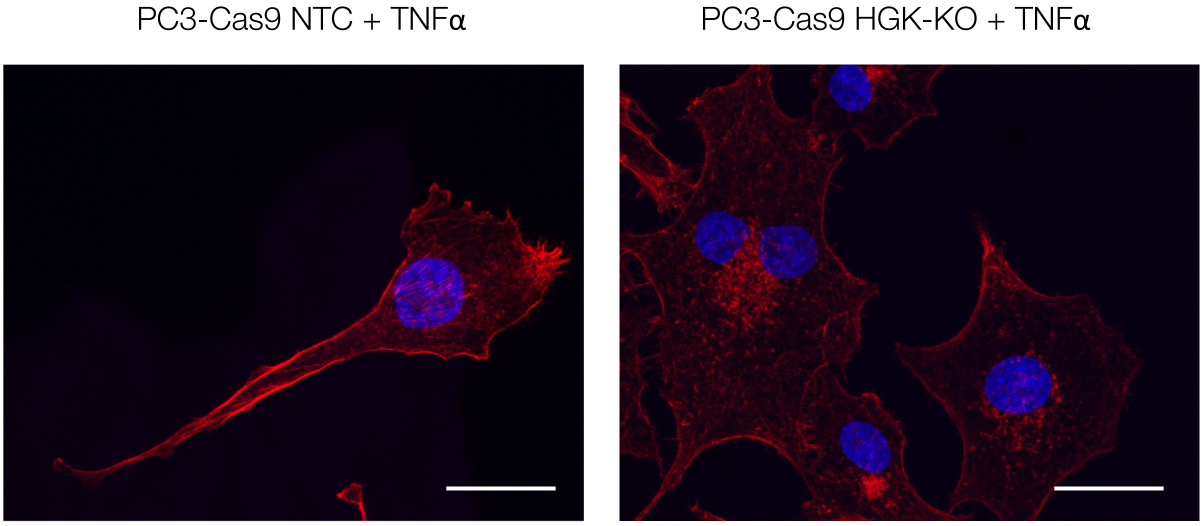

Supplementary Figure 5: Pictures of migratory PC3 cells Immuno-fluorescence microscopy images of phalloidin staining (*red*) in NTC and HGK depleted PC3 cells. Cell nuclei were stained with DAPI (*blue*). Scale bars: 50 µm. Images are maximum intensity projections taken in a LSCM microscope.

Supplementary Figure 6: Uncropped images Figure 1.

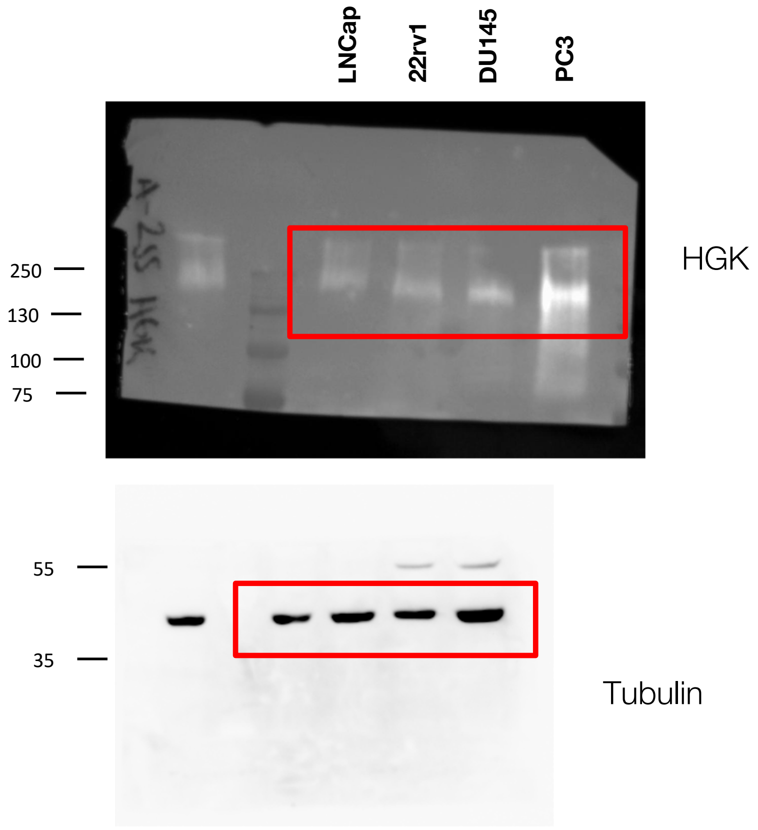

Supplementary Figure 7: Uncropped images Figure 2.

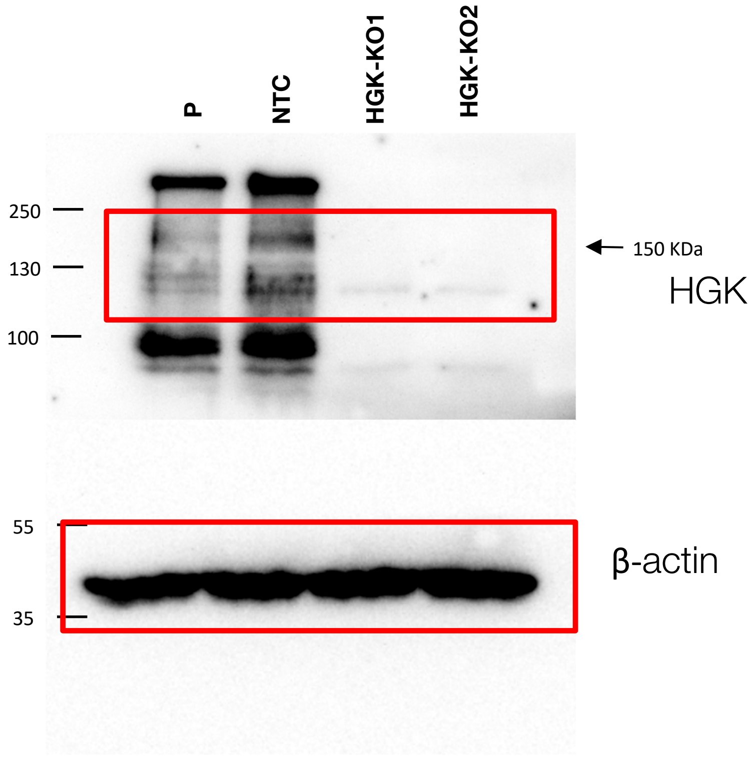

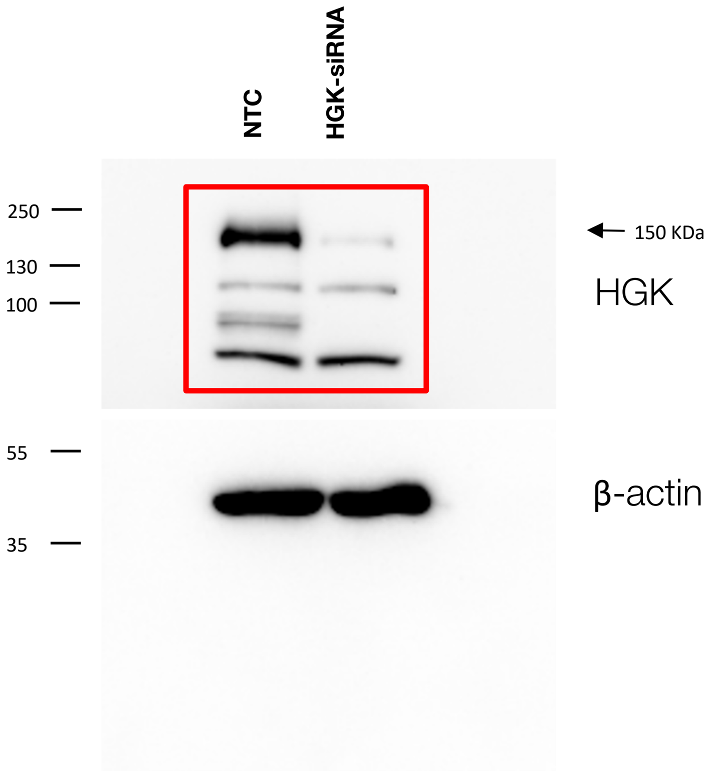

Supplementary Figure 8: Uncropped images Figure 5.

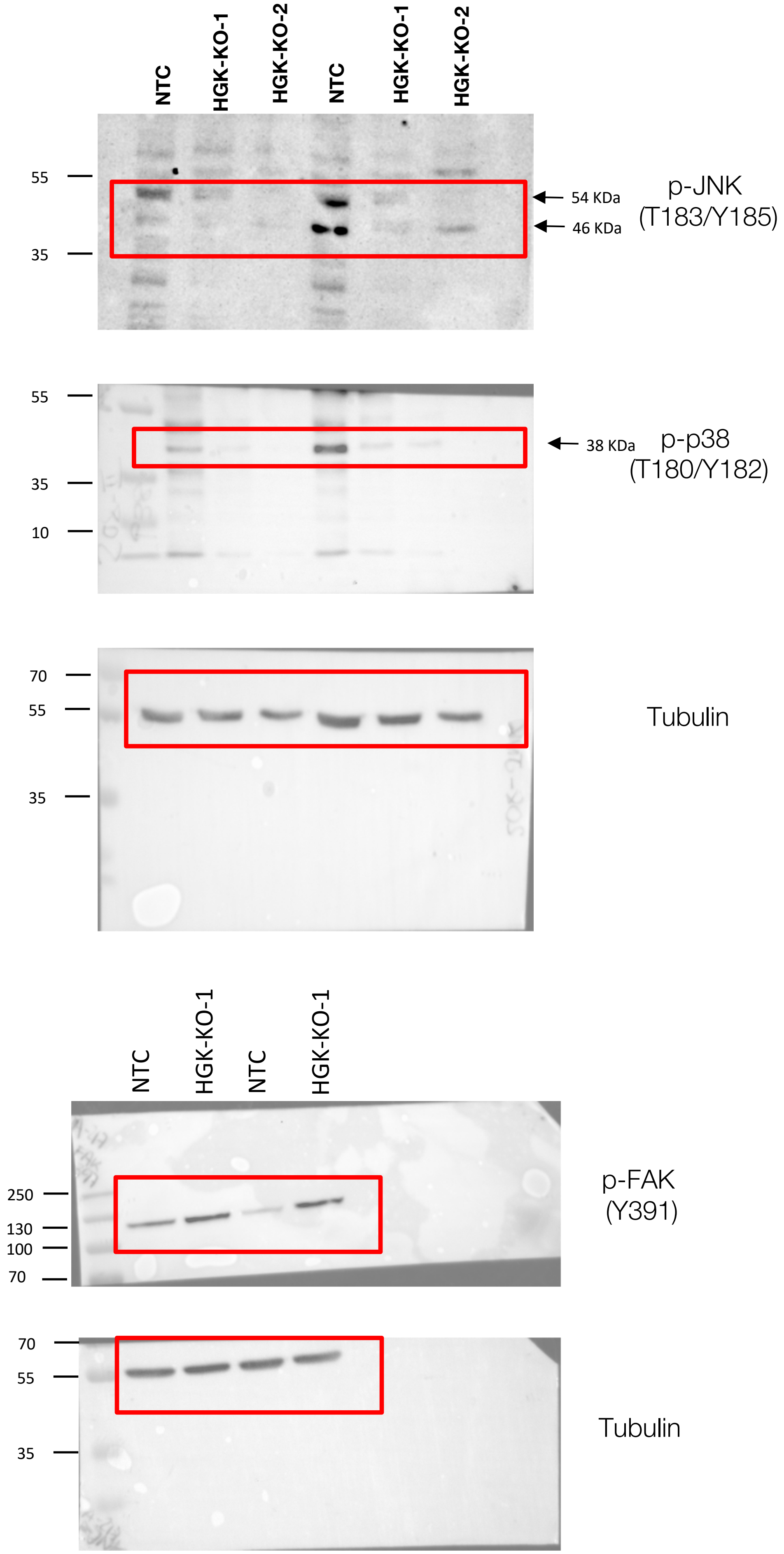

Supplementary Figure 9: Uncropped images supplementary Figure 3.

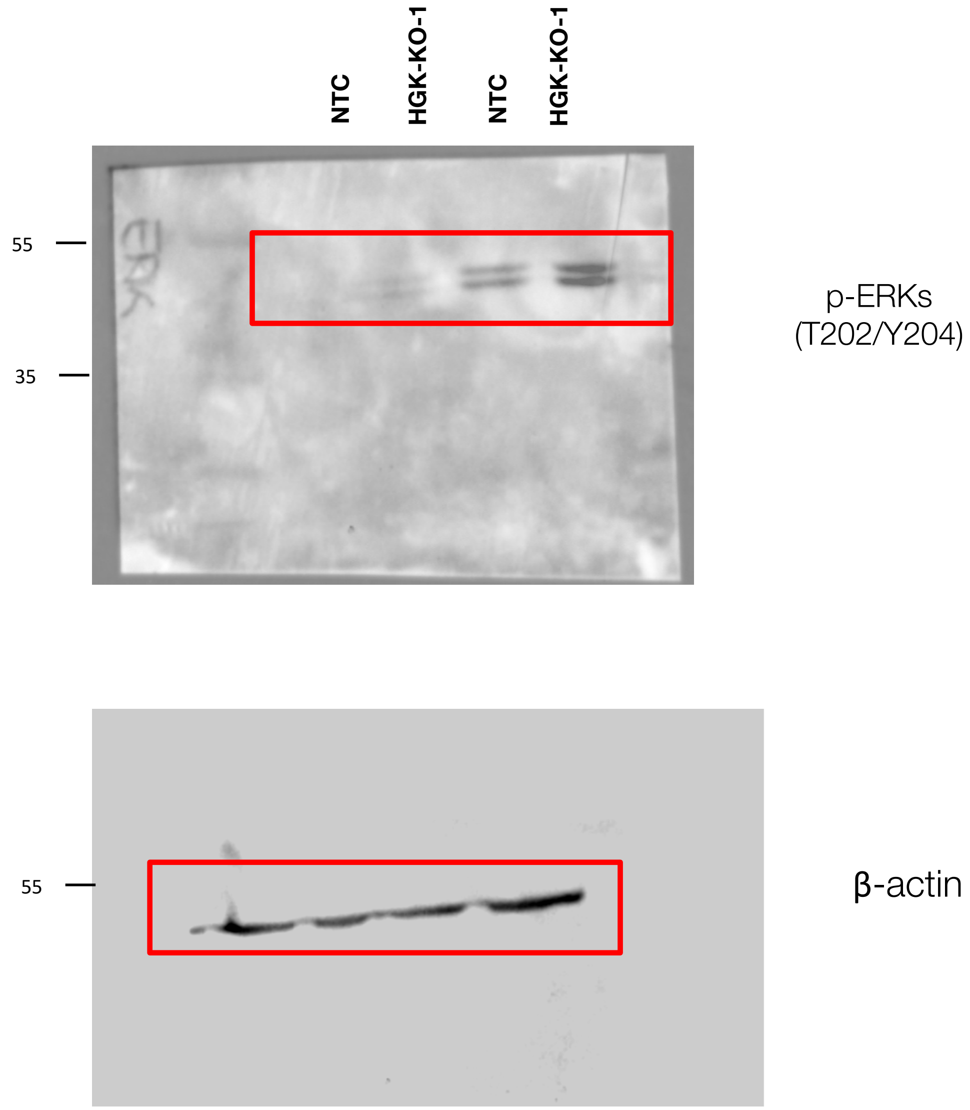

Supplement: Supplementary file 1 — Supplementary Information. [file 41598_2021_91292_MOESM1_ESM.pdf]
